# Supplementary material for: Nucleolar fibrillarin is an evolutionarily conserved regulator of bacterial pathogen resistance
Source: Nat Commun. 2018 Sep 6;9:3607. doi: 10.1038/s41467-018-06051-1 (PMC6127302; doi:10.1038/s41467-018-06051-1)
Supplement: Supplementary file 1 — Supplementary Information [file 41467_2018_6051_MOESM1_ESM.pdf]

Nucleolar Fibrillarin is an evolutionarily conserved regulator of bacterial pathogen resistance

Tiku et. al.

Supplementary Figure 1

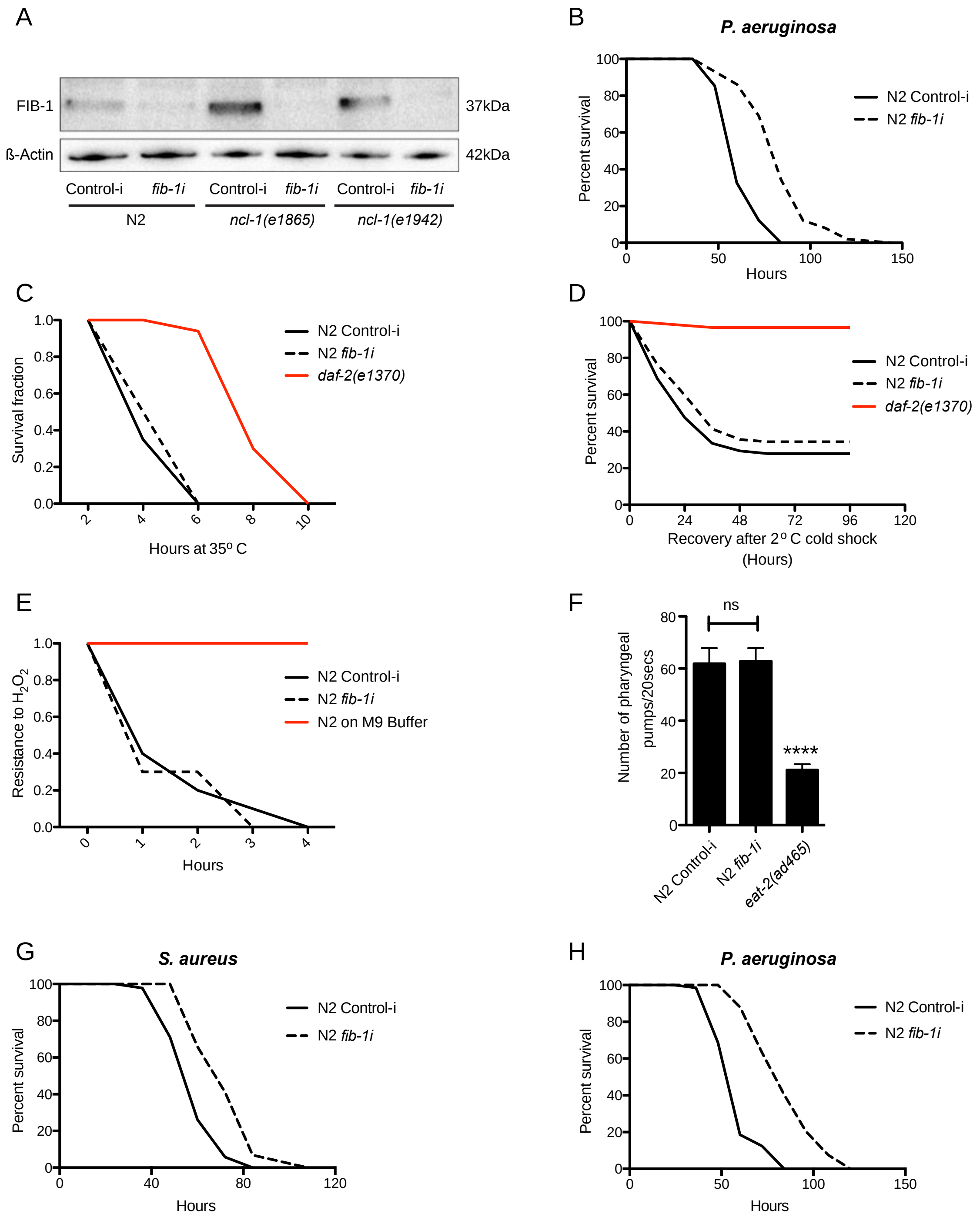

Supplementary Fig. 1. *fib-1*/Fibrillarin specifically regulates bacterial infection resistance in *C. elegans*. (A) *fib-1* RNAi from larval stage L3 up to day 1 adulthood (~ 30 hours) is sufficient to reduce the levels of FIB-1 protein in wildtype N2 and *ncl-1* mutants. (B) *fib-1* RNAi significantly enhances *P. aeruginosa* infection tolerance in wildtype N2 worms (P<0.0001, log-rank test). Survival experiments were performed three times independently. (C,D,E) *fib-1* RNAi does not significantly alter heat tolerance, cold shock recovery, or oxidative stress tolerance in wildtype N2 worms. (F) *fib-1* RNAi does not affect pharyngeal pumping rate. Error bars represent mean ± s.d. ****P<0.001, unpaired t-test. (G,H) Wildtype N2 worms cultured on plates completely covered with *S. aureus* and *P. aeruginosa* lawns display enhanced survival upon *fib-1* knockdown (P<0.0001, log-rank test).

Supplementary Figure 2

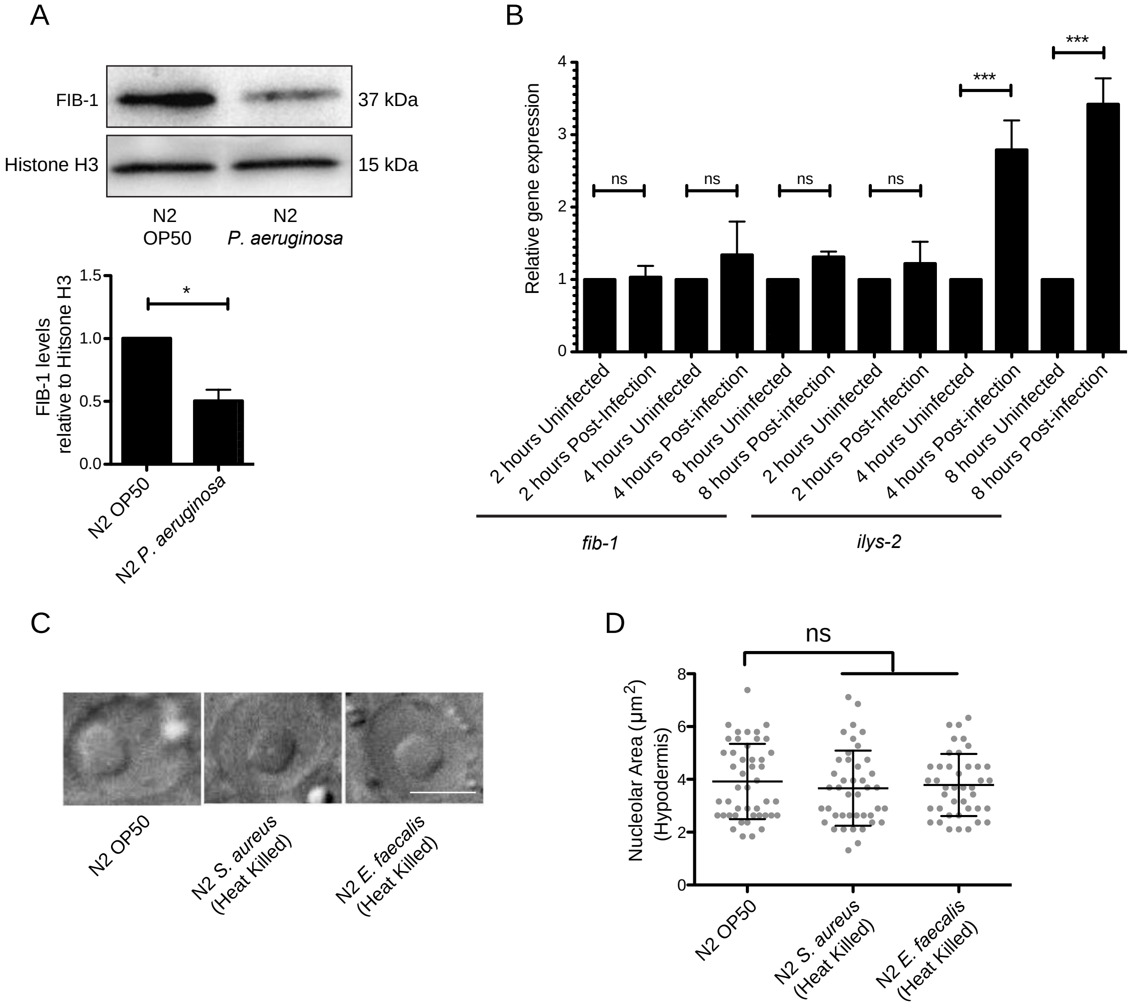

Supplementary Fig. 2. Bacterial infection reduces nucleolar size and FIB-1/Fibrillarin levels. (A) FIB-1 levels are significantly reduced in wildtype N2 after *P. aeruginosa* infection. Error bar represents mean ± s.e.m. of three independent biological replicates. *P<0.05, unpaired t-test (B) 2, 4 and 8 hour *S. aureus* infection does not change the expression levels of *fib-1. ilys-2* levels are increased significantly after 4 and 8 hours of infection and serves as a positive control for the experiment. Error bars represent mean ± s.e.m. from three independent biological replicates ***P<0.001, one-way ANOVA. (C,D) Treatment with heat-killed *S. aureus and E. faecalis* does not alter nucleolar size of hypodermal cells in wildtype N2 worms. Error bars represent mean ± s.d. ns non-significant, unpaired t-test. Scale bar represents 5 μm

Supplementary Figure 3

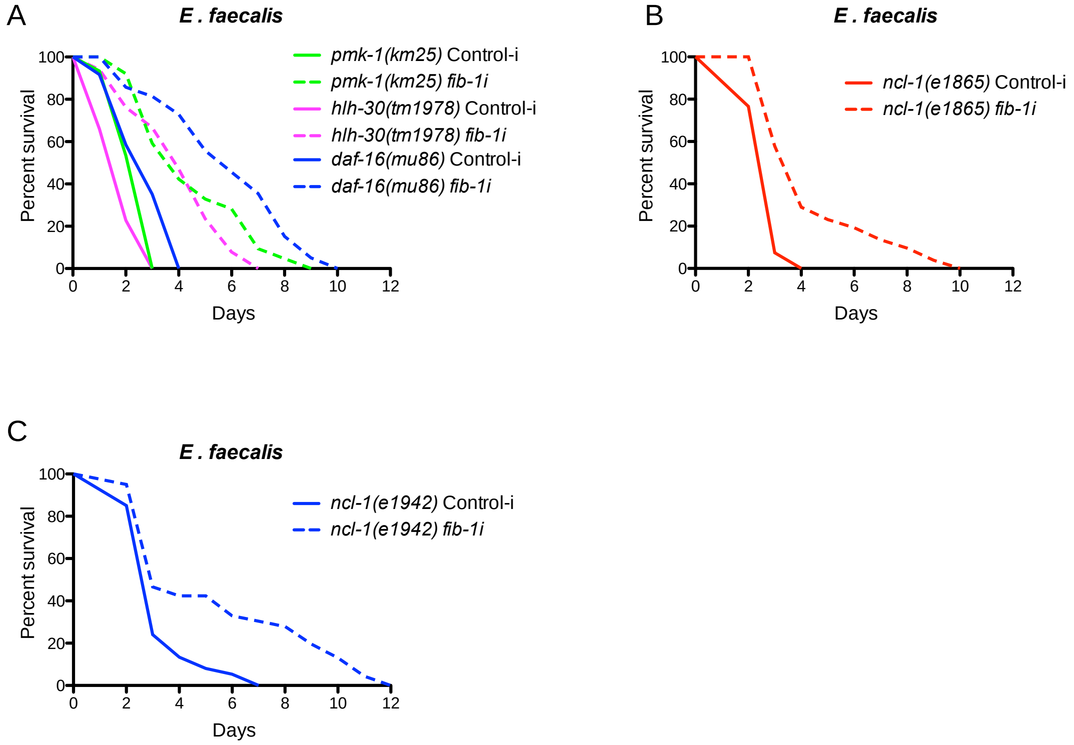

Supplementary Fig. 3. *fib-1*/Fibrillarin reduction improves resistance of infection sensitive mutants. (A,B,C) *fib-1* RNAi significantly improves the survival of infection sensitive *pmk-1(km25)* (P<0.0001, log-rank test), *hlh-30(tm1978)* (P<0.0001, log-rank test), *daf-16(mu86)* (P<0.0001, log-rank test) and *ncl-1(e1865* and *e1942*) mutants (P<0.0001, log-rank test) upon *E. faecalis* infection. The experiments were performed three times independently.

Supplementary Figure 4

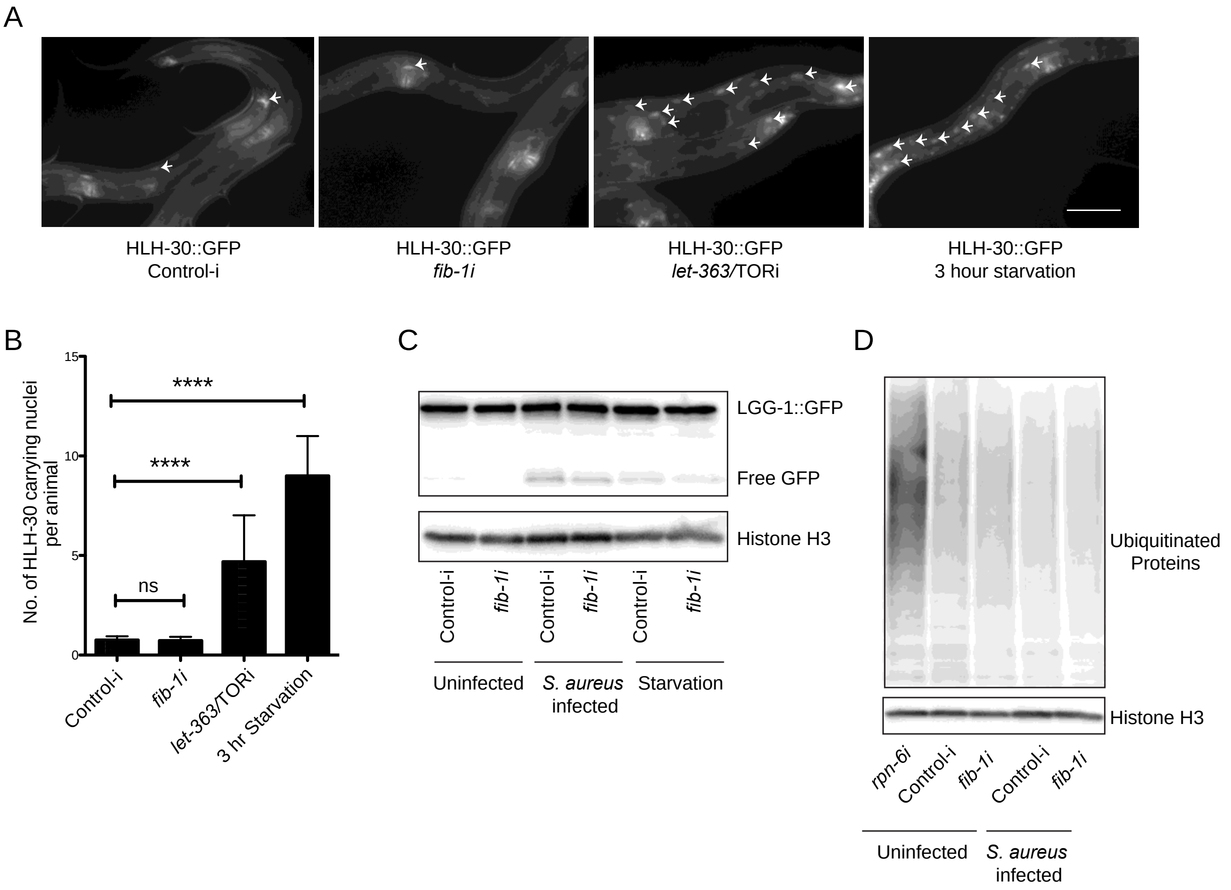

Supplementary Fig. 4. *fib-1/*Fibrillarin reduction does not influence autophagic and proteasomal degradation. (A,B) *fib-1* RNAi does not affect HLH-30 sub-cellular localization while *let-363/*TOR RNAi and 3 hour starvation significantly induce HLH-30 nuclear localization. White arrows show the nuclei with HLH-30::GFP. Error bars represent mean ± s.d. ****P<0.001, unpaired t-test. (C) Western blots showing the cleavage of LC3/LGG-1::GFP in wildtype and *fib-1* RNAi worms upon infection with *S. aureus.* Infection induced the appearance of free GFP band suggestive of enhanced autophagy. Starvation served as a positive control in the experiment. (D) Western Blot showing total ubiquitinated protein levels probed by anti-Ubiquitin antibody. RPN6 served as a positive control for the experiment. Scale bar represents 100 μm.

Supplementary Figure 5

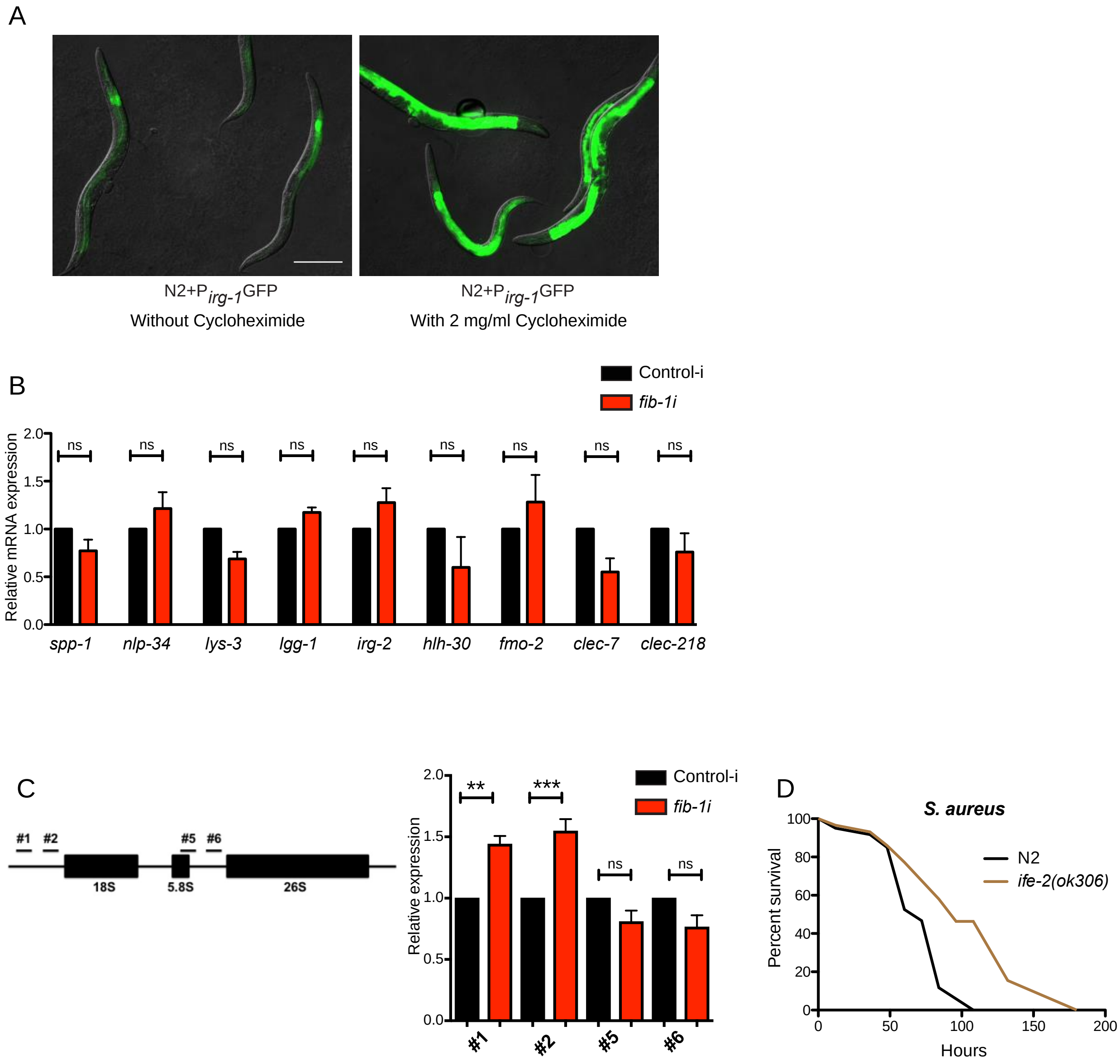

Supplementary Fig. 5. *fib-1*/Fibrillarin reduction induces translation suppression. (A) $P_{irg\text{-}1}$GFP reporter is strongly induced by a 3-hour Cycloheximide (2mg/ml) treatment. (B) *fib-1* RNAi does not significantly change the expression of infection related genes. Error bars represent mean ± s.e.m. from three independent biological replicates, ns non-significant one-way ANOVA. (C) *fib-1* RNAi increases the levels of pre-rRNA species. The primers were designed based on a recent study[47]. The illustration depicts the primer binding sites. Results were normalized to *snb-1* which

served as the housekeeping control for qPCR. Error bars represent mean ± s.e.m. from three independent biological replicates, one-way Anova was used for statistics (D) *ife-2(ok306)* is significantly longer-lived than wildtype N2 worms upon *S. aureus* infection (P=0.0034, log-rank test). The experiments were performed three times independently. Scale bar represents 200 μm.

Supplementary Figure 6

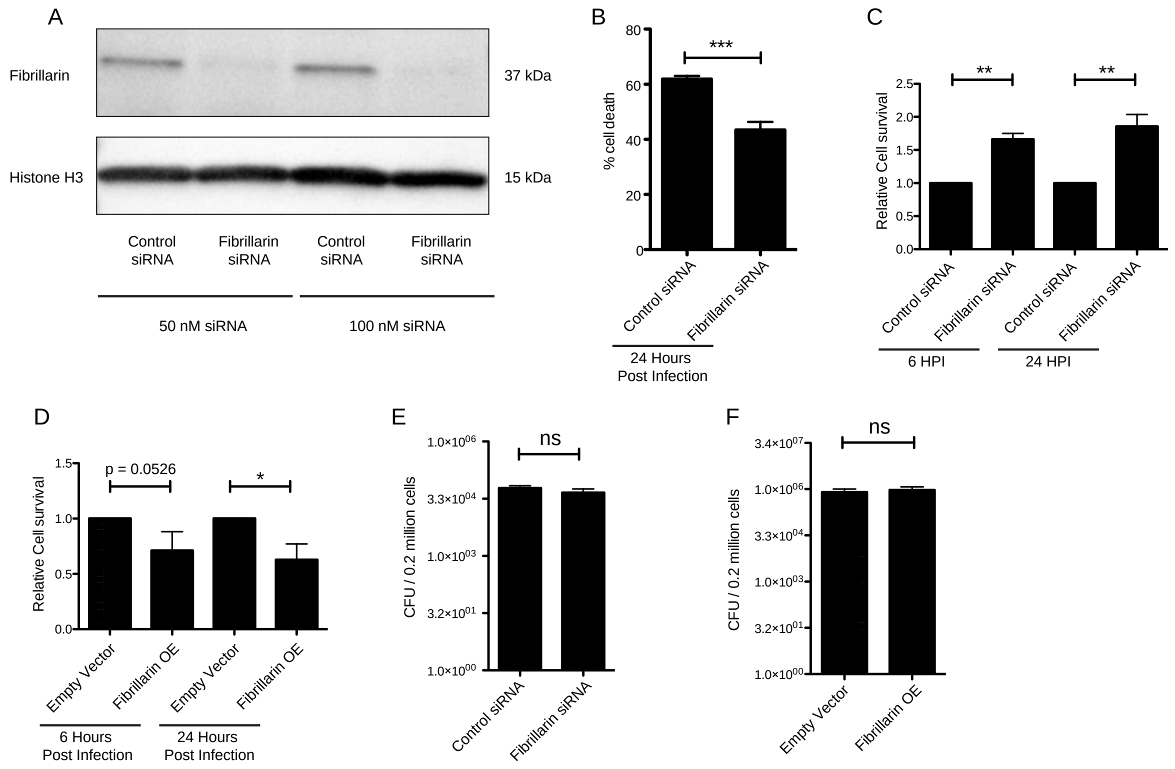

Supplementary Fig. 6. Fibrillarin reduction improves cell survival upon bacterial infection. (A) 50 and 100 nM Fibrillarin siRNA sufficiently knocks down the levels of Fibrillarin protein in HeLa cells. (B) Fibrillarin siRNA significantly reduces infection induced cell death as measured by LDH cytotoxicity assay (***P<0.001, unpaired t-test, error bars represent mean ± s.e.m. from three independent biological replicates). (C) Fibrillarin siRNA improves cell survival relative to control siRNA after 6 and 24 hours of *S. aureus* infection in HeLa cells as assayed by trypan blue staining. Error bars represent mean ± s.e.m., from three independent biological replicates, one-way ANOVA was used for statistics. (D) Fibrillarin over-expression reduces cell survival relative to control (empty vector) after 6 and 24 hours of *S. aureus* infection in HeLa cells. Error bars represent mean ± s.e.m from three independent biological replicates *P<0.05, one-way ANOVA. (E,F) Fibrillarin siRNA and over-expression do not alter bacterial uptake in HeLa cells, relative to respective controls, as measured by CFU analysis. Error bars represent mean ± s.d. from three independent biological replicates, ns non-significant, unpaired t-test.

Supplementary Figure 7

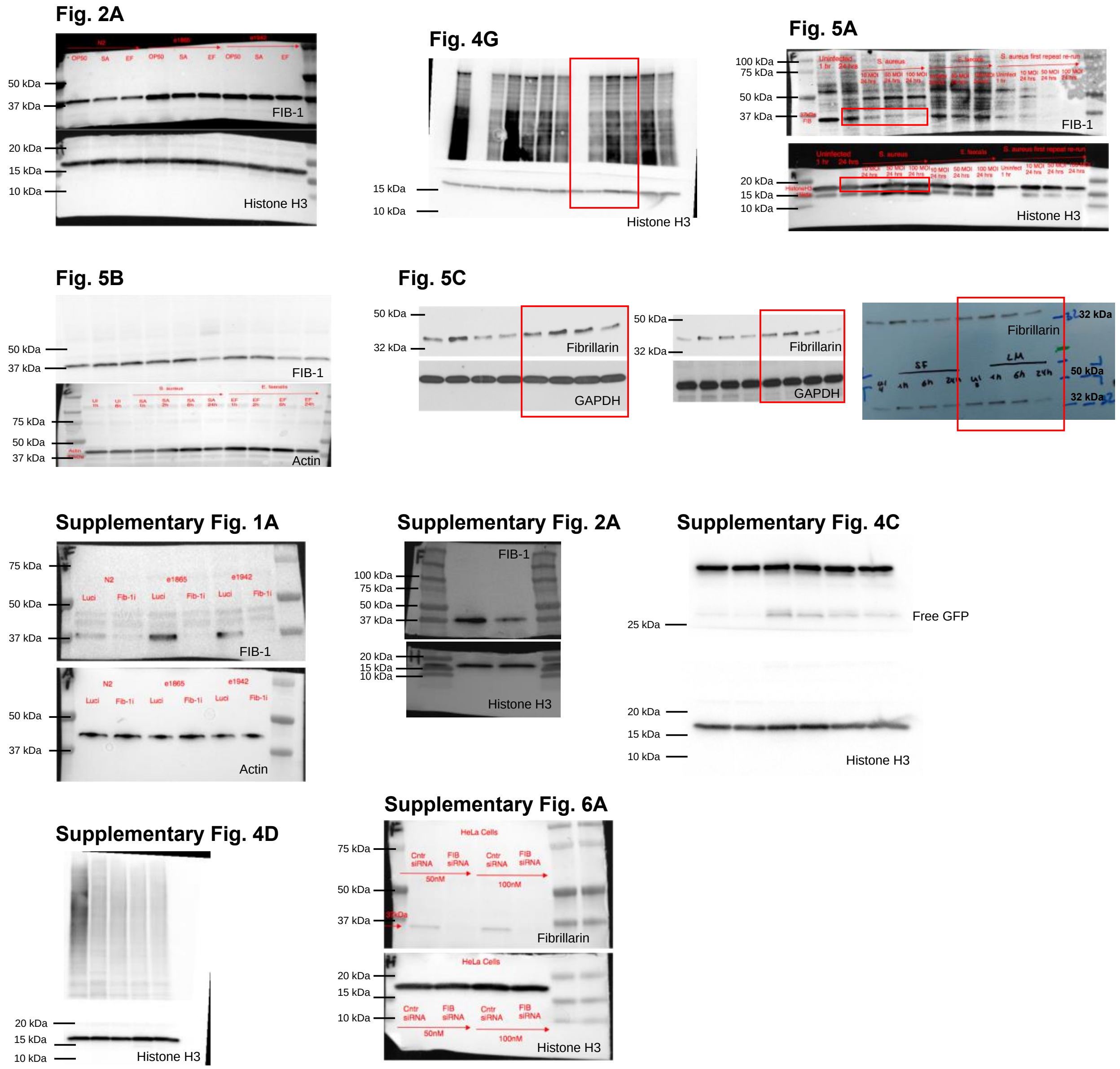

Supplementary Fig. 7. Uncropped versions of all the western blots shown in the main and supplementary figures.

Supplementary Table 1: Primers used for qRT-PCR

Target	Forward	Reverse
fib-1	CAAACGTTGTCCCAATTGTCG	GGAAGTTTTGGGCATTGAGAG
ilys-2	GTTGGATCGCTTTCTTGTGG	CGTCAGCACATCTCTTCCAG
irg-1	TGATCTTGTTCCGTACCCATG	ATCCTCTCCAGTTTCGTTCATC
spp-1	GGTGTTTTCTGTGATGTCTGC	ATAGTCCAGCAAAGAGTTCCG
nlp-34	TCATCGCTTGCCTGTTGG	CATGGGCGGTAGTATGGG
lys-3	CCAAGATATGATTAGAAGTGCGAAG	ACTAAACGTGTTCCAGCCTC
lgg-1	ACCCAGACCGTATTCCAGTG	ACGAAGTTGGATGCGTTTTC
irg-2	TGTTCGACGAGTTTTACTTCCG	CAATTGTGCCTTCAGTTTTCATG
hlh-30	GGCAGCGACAAAATTCACAG	TCATCTTCCATGCCCATGAG
fmo-2	TGCCAAACAAGTCTACCTAGTC	TGTAGAGTGAGAAGAAACGCG
clec-7	TGTTTATGGGACGATTCGACG	TCCTGTCAATGCACCTTGTAC
clec-218	GTTGGCAAGTGAAGGAAATGG	TGATATTTACGAGGACAGAAGCAG
#1 (pre-rRNA)	CTGTGTTTACACCCGAATGATTCTAG	CTAATCGTGAGATGGGACACTCATACA
#2 (pre-rRNA)	CGCAGACATATAGTCTAGCGAG	GATCCATAGATATTGCTGATGATTC
#5 (pre-rRNA)	AACGCATAGCACCAACTG	TCCGAAGAGAAGCCTAAG
#6 (pre-rRNA)	AATACTGGGATTCGTCTA	GAGTTCAGGTTGAGATTAG
snb-1	GAATCATGAAGGTGAACGTGG	CCAATACTTGCGCTTCAGGG
